# Regulation of immune response against third-stage *Gnathostoma spinigerum* larvae by human genes

**DOI:** 10.3389/fimmu.2023.1218965

**Published:** 2023-08-03

**Authors:** Pattarasuda Puasri, Wilanee Dechkhajorn, Paron Dekumyoy, Tippayarat Yoonuan, Sumate Ampawong, Onrapak Reamtong, Usa Boonyuen, Surachet Benjathummarak, Yaowapa Maneerat

**Affiliations:** ^1^ Department of Tropical Pathology, Faculty of Tropical Medicine, Mahidol University, Bangkok, Thailand; ^2^ Department of Helminthology, Faculty of Tropical Medicine, Mahidol University, Bangkok, Thailand; ^3^ Department of Molecular Tropical Medicine and Genetics, Faculty of Tropical Medicine, Mahidol University, Bangkok, Thailand; ^4^ Center of Excellence for Antibody Research (CEAR), Faculty of Tropical Medicine, Mahidol University, Bangkok, Thailand

**Keywords:** *Gnathostoma spinigerum*, gene expression, extracellular vesicles, immune evasion, pathogenesis, gnathostomiasis, third stage *G. spinigerum* larvae

## Abstract

**Background:**

Gnathostomiasis is an important zoonosis in tropical areas that is mainly caused by third-stage *Gnathostoma spinigerum* larvae (*G. spinigerum* L3).

**Objectives:**

This study aimed to prove whether *G. spinigerum* L3 produces extracellular vesicles (EVs) and investigate human gene profiles related to the immune response against the larvae.

**Methods:**

We created an immune cell model using normal human peripheral blood mononuclear cells (PBMCs) co-cultured with the larvae for 1 and 3 days, respectively. The PBMCs were harvested for transcriptome sequencing analysis. The EV ultrastructure was examined in the larvae and the cultured medium.

**Results:**

Extracellular vesicle-like particles were observed under the larval teguments and in the pellets in the medium. RNA-seq analysis revealed that 2,847 and 3,118 genes were significantly expressed on days 1 and 3 after culture, respectively. The downregulated genes on day 1 after culture were involved in pro-inflammatory cytokines, the complement system and apoptosis, whereas those on day 3 were involved in T cell-dependent B cell activation and wound healing. Significantly upregulated genes related to cell proliferation, activation and development, as well as cytotoxicity, were observed on day 1, and genes regulating T cell maturation, granulocyte function, nuclear factor-κB and toll-like receptor pathways were predominantly observed on day 3 after culture.

**Conclusion:**

*G. spinigerum* L3 produces EV-like particles and releases them into the excretory-secretory products. Overall, genotypic findings during our 3-day observation revealed that most significant gene expressions were related to T and B cell signalling, driving T helper 2 cells related to chronic infection, immune evasion of the larvae, and the pathogenesis of gnathostomiasis. Further in-depth studies are necessary to clarify gene functions in the pathogenesis and immune evasion mechanisms of the infective larvae.

## 1 Introduction

Human Gnathostomiasis is a zoonosis caused by third-stage *Gnathostoma* spp. larvae (L3). At least five of the 12 species in the genus (*G. binucleatum*, *G. doloresi*, *G. hispidum*, *G. nipponicum* and *G. spinigerum)* cause human disease. The species most frequently found in humans and most widely distributed around the world is *G. spinigerum. G. binucleatum* is found in the Americas (1). Sporadic cases caused by *G. doloresi, G. hispidum*, and *G. nipponicum* have been documented in Asia. *G. spinigerum* is the most common cause of the disease worldwide, particularly in Southeast Asia and Thailand (1). The disease can develop through 3 modes of transmission including oral, transplacental and skin wounds. Among the human cases, more than 90% caused by ingestion of raw or undercooked meat of intermediate hosts, such as fish, frogs, snakes or poultry, which contains *G. spinigerum* L3.

Epidemiological studies have revealed that *Gnathostoma* spp. is distributed worldwide. About 5,000 cases of human gnathostomiasis have been reported globally. The first case was described in Thailand, in 1889. The disease is endemic to Japan and Thailand, and has been reported sporadically in many countries around the world. Three thousand, one hundred and eighty-two (3,182) cases of human gnathostomiasis were detected in Japan between 1911 and 1995. In Thailand, 1,079 cases of human gnathostomiasis have been reported. The seroprevalence of Gnathostoma in humans was 62.5% (531/849) in Bangkok, Thailand, between 2000 and 2005. The high prevalence of gnathostomiasis among this population might be due in part to the local custom of eating raw fish. The first case of human gnathostomiasis in China was reported in Xiamen, Fujian Province, in 1919. Eighty-three cases (of which 80 were caused by *G. spinigerum*, two by *G. hispidum* and one by *G. doloresi*) were reported between 1918 and 2014, mostly in southern and eastern China (reviewed in (2)).

Although the infective larvae cannot develop into the adult stage in humans, they migrate within the host’s body and provoke an inflammatory reaction and associated clinical symptoms. Cutaneous gnathostomiasis is relatively common, and is characterised by intermittent migratory swelling, usually in the trunk and upper limbs. Visceral larval migrans is a more serious condition but less frequently found. It occurs when the L3 (mostly *G. spinigerum*) migrate throughout various organs, such as the eyes, ears, breasts, lungs, gastrointestinal tract, thoracic spinal cord, genito-urinary system and central nervous system (CNS). Human cerebral gnathostomiasis with neurological manifestations, such as eosinophilic encephalomyelitis, intracranial haemorrhage and tract haemorrhage, can cause sudden death (1–3). Our current understanding of the host immune response and the larval immune evasion strategies remains unclear.

Previous studies of helminthic diseases reported the association of cutaneous and visceral migrans with excretory-secretory products (ESPs) from infective larvae (4–6). ESPs are composed of many essential molecules, such as protease and hyaluronidase, responsible for tissue invasion, protein degradation and anticoagulation inhibition, as well as acting as anti-inflammatory agents and modulators of the host immune response or causing the pathology of helminthic diseases (5–10). Until now, the role of *G. spinigerum* L3 ESPs in larvae migrans, immune evasion and pathogenesis has not been fully elucidated.

Extracellular vesicles (EVs) are membrane-enclosed structures secreted by various cell types. During their biogenesis, EVs may selectively capture cell-specific proteins, lipids, RNAs or even DNA, which may become a part of the EV membrane or molecular cargo (11). EVs play key roles in both homeostasis and disease pathogenesis by participating in intercellular signalling and communication. Several parasite species, including the adult and larval stages of some helminths, produce EVs and release them into ESPs (12). EVs are classified into three major subtypes: exosomes, microvesicles (MVs) and apoptotic bodies (11). EVs are found in body fluids, such as blood and urine. It was reported that EVs modulate host cells to enhance pathogenesis and/or inhibit immune responses. Due to the resemblance of the composition of EVs with the parental cell, circulating EVs have attracted considerable interest as a potential source of undiscovered biomarkers (12). Several recent intensive studies have revealed that parasite EVs are composed of bioactive contents, including proteins, microRNA (miRNA), noncoding RNA, and lipid. Nowadays, the mechanisms of EV uptake and cargo delivery into the cytosol of target cells in the infected host remain incompletely clarified. Previous studies demonstrated that bioactive molecules secreted by parasitic nematodes, packaged in exosomes, function as cell-to-cell effectors in the host-parasite interaction and immunomodulator in immune cells. For example, proteins and small RNA species in EVs secreted by *Heligmosomoides polygyrus* (*H. polygyrus)* alter gene expression in host cells and suppress innate immune responses, and reduce eosinophilia in the lungs of mice. *O. viverrini* EVs on human cholangiocytes found that they enhanced cell proliferation and induced the development of cholangiocarcinoma in liver fluke-infected patients. *Brugia malayi* EVs contain migration inhibitory factor for macrophage activation. (reviewed in (13)). There is currently no evidence to prove whether *G. spinigerum* L3 produce EVs that are released into ESPs. Moreover, in-depth bioinformatics studies of host immunity against infective *G. spinigerum* L3 are crucial to understand the pathogenesis of human gnathostomiasis. Such studies can be performed using next-generation sequencing (NGS) analysis, which efficiently sequences the whole genome and detects abnormalities, including copy number changes and variations. Compared with traditional DNA sequencing, NGS is cheaper, has a faster turnaround time and requires a smaller amount of DNA (14).

The aims of this study were two-fold: 1) to prove whether *G. spinigerum* L3 produces EVs and 2) to explore human gene profiling related to the immune response to *G. spinigerum* L3. We co-cultured peripheral blood mononuclear cells (PBMCs) obtained from healthy buffy coats with live *G. spinigerum* L3 for 3 days. On days 1 and 3 after culture, the PBMCs were collected for RNA extraction and subsequent transcriptomic analyses using NGS. Gene profiling of human PBMC’s response against *G. spinigerum* L3 will provide a better understanding of gnathostomiasis pathogenesis and may be advantageous for future studies of immunotherapeutic strategies.

## 2 Materials and methods

### 2.1 Study design and subjects

**Figure 1:**
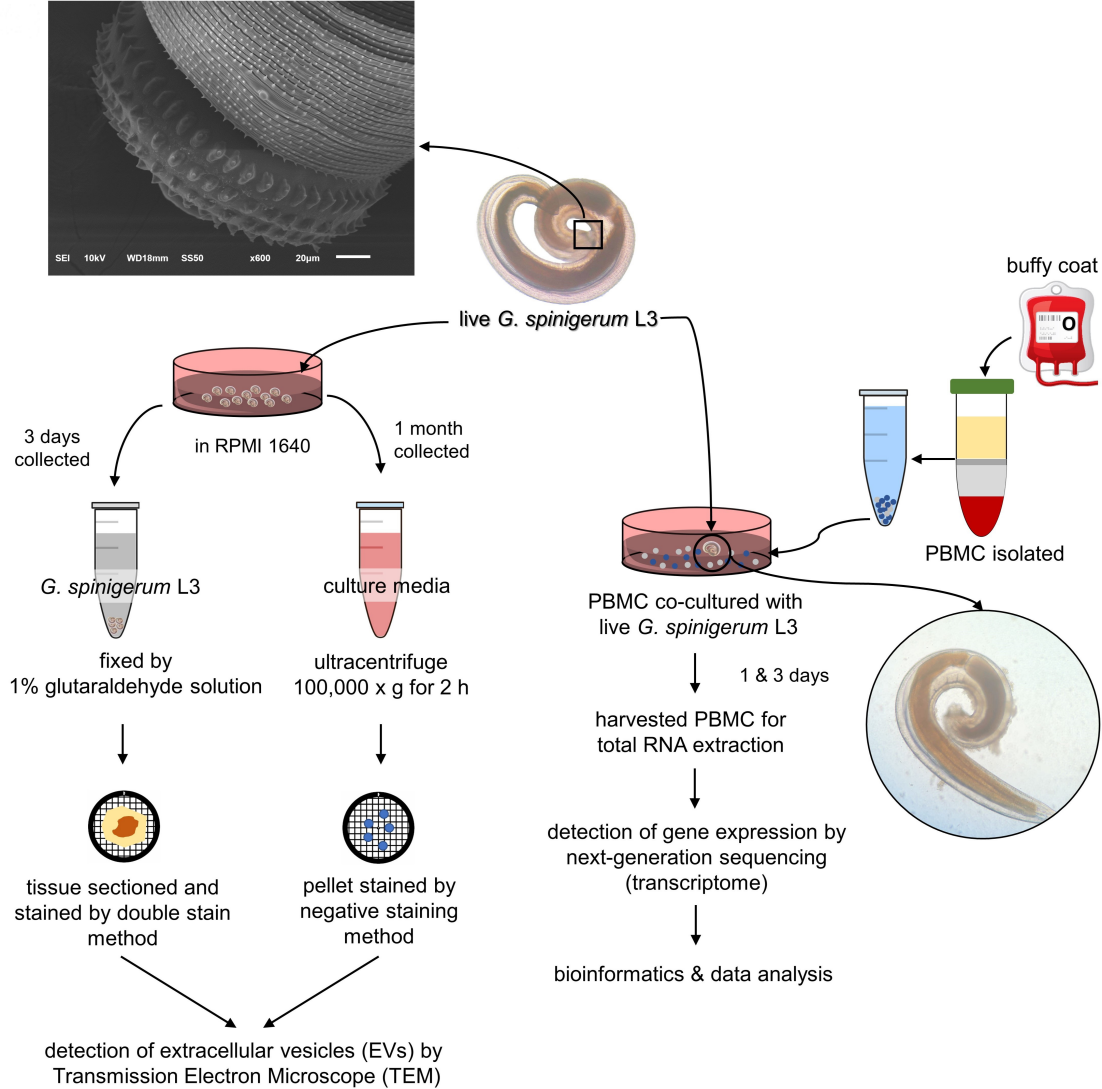
Experimental design was divided into 2 parts according to aims of the study. 1) to prove whether third stage *G. spinigerum* larvae (*G. spinigerum* L3) could produce extravascular vesicles (EVs), live *G. spinigerum* L3, obtained from eel livers, were cultured for excretory secretory product (ES) collection. Extracellular vesicles (EVs) in the ultra-centrifuged pellets of the collected cultured medium and in larvae were processed and examined by transmission electron microscopy; and 2) to inspect human gene profiling related to the immune response to *G. spinigerum* L3, peripheral blood mononuclear cells (PBMCs) obtained from healthy buffy coats were co-cultured with live *G. spinigerum* L3 for 3 days. At day 1 and 3 after cultivation, the cultured PBMCs were collected for RNA extraction and further performed next generation sequencing based transcriptomic analyses. Gene profiling and corresponding proteins were selected and discussed their roles and interplays in immune response against the larvae and possible pathogenesis of human gnathostomiasis.

This study was performed at the Faculty of Tropical Medicine, Mahidol University. The study was approved by 1) the Ethics Committees of the Faculty of Tropical Medicine, Mahidol University (MUTM 2021-069-01), 2) the Thai Red Cross Society, Bangkok, Thailand (NBC 21/2021), and 3) Faculty of Tropical Medicine- Animal Care and Use Committee (FTM-ACUC 008/2020E). Normal PBMC were separated from healthy buffy coats provided by the Thai Red Cross Society. These PBMCs were used as a human immune cell model for the present study. The experimental design is summarised in **Figure 1**.

### 2.2 *G. spinigerum* L3 preparation from eel liver

*G. spinigerum* L3 were obtained from the livers of naturally infected eels by 1% acid -pepsin digestion (15). After treatment, the larvae in the eel liver suspension were collected under a dissecting microscope, washed several times with normal saline solution, and finally with sterile distilled water before use.

### 2.3 Examination of parasite and EVs morphology

#### 2.3.1 Examination of EVs in ESP from the *G. spinigerum* L3 cultivation

To investigate whether EVs were found in the ES product, a pellet was collected from ultracentrifugation of the pooled 7-day *G. spinigerum L3* cultured media and processed as described previously (16). The pellets were fixed with 2.5% glutaraldehyde in PBS for 1 hour (h), then washed in PBS. Ten microliters of pellet suspension in sucrose phosphate buffer (SPB) pH 7.4 was dropped on Formvar grids. After negative staining with uranyl acetate, the morphology and size of EV-like particles were observed under a transmission electron microscope (TEM) (Hitachi; model HT7700, Japan).

#### 2.3.2 Examination of EVs in *G. spinigerum* L3 by TEM

To prove that *G. spinigerum* L3 could produce EVs, day 3-cultured larvae were harvested and processed as described previously (17). Briefly, the larvae were fixed with 2.5% glutaraldehyde for at least 1 h, then washed 3 times for 10-15 minutes (min) each in sucrose phosphate buffer (SPB) pH 7.4. The larvae were then soaked in 1% osmium tetroxide for 1 h, then washed 3 times for 10-15 min each with SPB. The larvae were dehydrated twice in graded ethanol for 15 min each. The larvae were then infiltrated with Epon (EMS, Hatfield, PA, USA) in acetone and then embedded in a capsule beam. After that, the embedded larvae were polymerized in a 60 °C incubator blocks for 72 h and then cut into 90–100-nm thick sections. The sections were negative-stained using uranyl acetate, and then observed under a TEM.

#### 2.3.3 Identification of *G. spinigerum* L3 by SEM

To examine the characteristics of *G. spinigerum* L3, five larvae were randomly collected and processed (18). The larvae were fixed with 2.5% glutaraldehyde for 1 h and then post-fixed with osmium tetroxide for 1 h. Samples were then dehydrated twice in graded ethanol for 10 min each. After dehydration, the samples were put into a critical point dryer (CPD) and then placed on an aluminum stub sputtered with gold in the coating unit (model K550, Emitech Ltd., Kent, England), and examined under a scanning electron microscope (model JSM-6610LV, JEOL Ltd., Tokyo, Japan).

### 2.4 Live *G. spinigerum L3* co-cultured with human PBMCs

3 ×10^6^ PBMCs (CD27-)/well in 6 well plates were co-cultured with one live *G. spinigerum* L3 in complete medium (RPMI1640 supplemented with 10% heat-inactivated fetal bovine serum) at 37°C in an atmosphere of 5% CO_2_. On days 0, 1, or 3 after incubation, cultured PBMCs were harvested, and washed 3 times with D-PBS (Thermo Fisher Scientific, Waltham, MA, USA) for RNA extraction. In this study, two independent experiments were performed in duplicate.

### 2.5 RNA extraction

Total RNA was extracted from PBMC pellets (15). Briefly, after washing, PBMC pellets were completely lysed with 1 ml of Trizol™ (Invitrogen, Carlsbad, CA, USA). Two hundred microliters of chloroform were added to the lysate, vortexed and incubated at room temperature for 10 min. Total RNA was extracted from each lysate using a phenol-chloroform method, as previously described (15, 19). RNA pellets were washed with 75% ethanol, dried and resuspended with sterile distilled water. Total RNA was measured by Qubit 4 Fluorometer (Thermo Fisher, Waltham, MA, USA). Samples were used for cDNA library construction.

### 2.6 cDNA library construction and sequencing by NGS technique

The procedure was performed as mentioned previously (20). The amount and quality of total RNA samples were determined before analysis. The integrity of the total RNA was assessed using an Agilent 2100 Bioanalyzer (Agilent Technologies, Santa Clara, CA, United States). Approximately 500 ng of the total RNA from each sample were used to create individually indexed strand-specific RNA-seq libraries using a TruSeq stranded mRNA library preparation kit (Illumina Inc., San Diego, CA, United States). Briefly, poly-A-containing mRNA molecules were captured using magnetic oligo (dT) beads, purified, and directed to cDNA synthesis. AMPure XP beads (Beckman Coulter Genomic, Atlanta, GA, United States) were used to separate the cDNA from the reaction mix. Indexing adapters were ligated to the cDNA, and all cDNA libraries were checked for quality using an Agilent 2100 Bioanalyzer and quantified using a fluorometer (DeNovix Inc., Wilmington, DE, United States). The indexed sequencing libraries were pooled in equimolar quantities and subjected to cluster generation and paired-end 2 × 75 nucleotide read sequencing on an Illumina NextSeq 500 sequencer. The sequencing process was carried out at Omics Sciences and Bioinformatics Center, Chulalongkorn University, Bangkok, Thailand.

### 2.7 Differential expression analyses of RNA-seq data and statistical methods

Bioinformatics analyses were performed according to the instructions from the manufacturers and as described previously (20). Briefly, the analyses comprised an initial quality check of the raw data files using FASTQC software (Bioinformatics Group, Babraham Institute, Cambridge, United Kingdom). Adapter and low-quality reads were depleted using Trimmomatic (21) http://www.usadellab.org/cms/index.php?page=trimmomatic. The filtered reads were aligned to a human reference genome using HISAT2 aligner software (Center for Computational Biology, Johns Hopkins University, Baltimore, MD, United States). StringTie (Center for Computational Biology, Johns Hopkins University) was used to assemble transcripts from RNA-seq reads that were aligned to the genome. Fold change ≥ 1, *p*-value < 0.05, and false discovery rate (FDR) < 0.05 was interpreted statistically significant. Gene profiling with fold change ± 4.0 were selected for further analyses in the current study. The gffcompare utility (22) (Center for Computational Biology, Johns Hopkins University, Baltimore, MD, United States), StringTie was used to discover a novel transcript. Gene Ontology (GO) and pathway enrichment analyses were done using a web-based bioinformatics tool DAVID (OmicX, Seine Innopolis, Le-Petit-Quevilly, France).

### 2.8 Correlate functions of some interesting genes to pathogenesis or immuno-evasion strategies of *G. spinigerum* L3 in Gnathostomiasis

Significant differential gene profiles expressed in *G. spinigerum* L3 co-cultured PBMCs on day 1 or day 3 were selected to explore properties and functions based on a reference database and linked to pathogenesis and/or immune-evasion strategies in human gnathostomiasis.

## 3 Results

### 3.1 Ultrastructural characteristics of *G. spinigerum* L3

The cultured larvae were examined under a scanning electron microscope (model JSM-6610LV, JEOL, Japan). They were identified as *G. spinigerum* L3, having morphological characteristics consistent with those mentioned previously (15, 23).

### 3.2 *G. spinigerum* L3 produces and releases EVs

**Figure 2:**
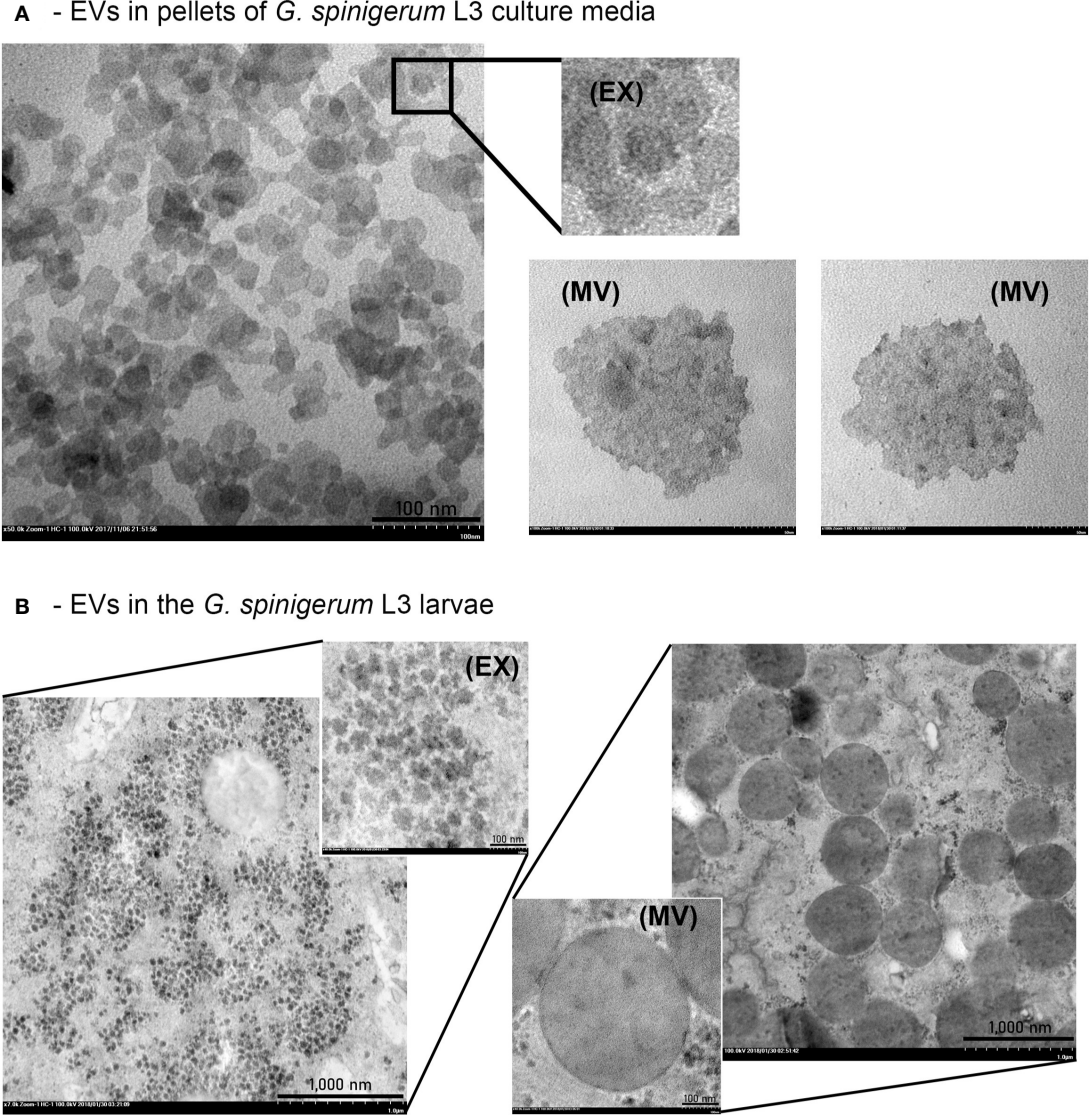
*G*. *spinigerum* L3 produce and release the EVs. Ultrastructural examination under transmission microscope: **(A)** The presence of EVs like vesicles in pellets of the larval cultured medium. Both exosome (EX) liked vesicles (size = 27.90 ± 1.75 nm), and microvesicle (MV)-like vesicles with small size (146.71 ± 13.76 nm) were observed, and **(B)** Some EX and MV-like vesicles were noted under cuticles of the larvae. The average sizes were 47.22 ± 8.17 nm, and 471.33 ± 136.70 nm, respectively.

The presence of EVs and typical morphology in the pellets of *G. spinigerum* L3 in the culture medium and under the larval teguments are shown in **Figures 2A, B**, respectively. EV-like vesicles were found in the pellets (**Figure 2A**). Both exosome-like vesicles (EX) (size = 27.90 ± 1.75 nm), small MV like vesicles (146.71 ± 13.76 nm) (MV) and large MV like vesicles (583.02 ± 136.03 nm) (pictures not shown) were observed. Examination of the larvae revealed the presence of some exosome- and MV-like vesicles under the cuticles (**Figure 2B**). The average sizes of the exosome and MV-like vesicles were 47.22 ± 8.17 nm (EX) and 471.33 ± 136.70 nm (MV), respectively. The double membranes of these vesicles were not obvious, but the vesicle sizes fell within the ranges of exosomes and MVs, respectively.

### 3.3 Gene expression selection from PBMCs co-cultured with *G. spinigerum* L3

**Figure 3:**
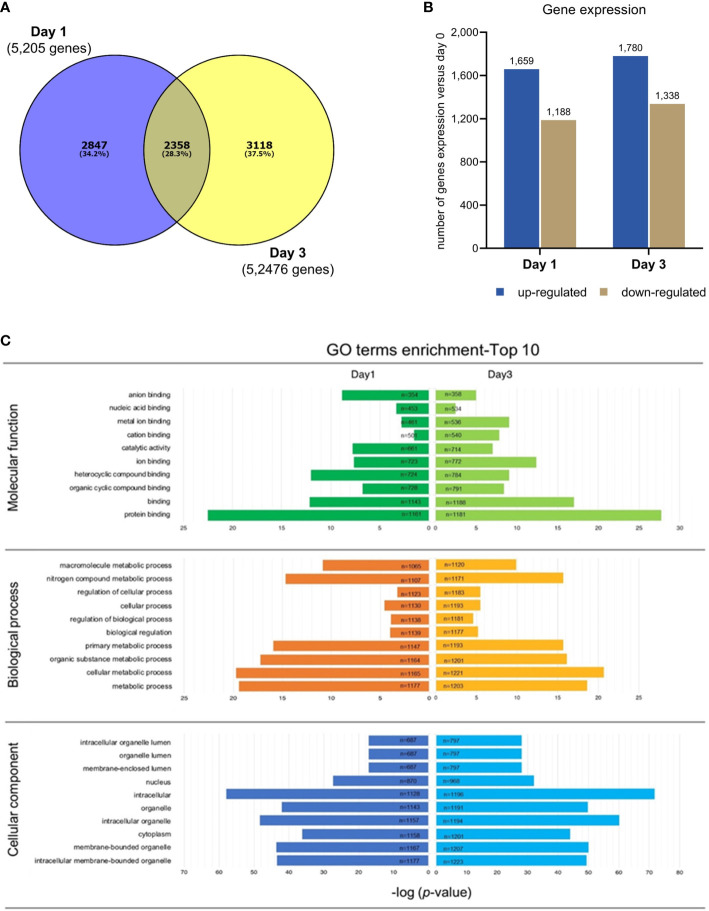
RNA-Seq analysis in PBMC co-cultured with *G*. *spinigerum* L3. **(A)** The venn diagram shows numbers of genes significantly expressed at day 1 (2,847 genes) or day 3 (3,118 genes), in comparison with those at day 0 of cultivation. These genes were further investigated. The 2,358 intersected genes were not focused in this study. **(B)** The bar graph indicates a number of up- and down regulated genes from 2,874 genes expressed at day 1, and 3,118 genes at day 3. **(C)** The illustration of gene ontology (GO) analysis. The GO classification considers 3 categories including molecular function, biological process, and cellular component. The most substantial enrichments (*p* < 0.05) in function and process. Expression and functions of genes at day1 and 3 of culture are shown in the left and right panels, respectively. Protein binding (n = 1161) was the most predominant term in “Molecular function”, both at day1 and day3. Metabolic process (n = 1177) was the top GO term identified in “Biological process”, while intracellular membrane (n = 1128) was the most predominant term in “Cellular component” at day 1. The enrichment of GO term at day 3 shows the same results as day 1, by which protein binding (n = 1181), metabolic process (n = 1171) and intracellular membrane (n = 1196) were the most abundant.

After RNA-seq analysis, the raw data files were exported and the data were sorted using a bioinformatics platform. In this study, only gene profiles that were significantly expressed on day 1 (2,847 genes) or day 3 (3,118 genes) after culture, in comparison with those expressed on day 0, were investigated further. There were 2,358 genes co-expressed on both days 1 and 3, meaning that they were expressed throughout the 3 days of culture (**Figure 3A**) and were thus not the focus of the present study. A number of up- and down-regulated genes from the 2,874 genes from day 1 and 3,118 genes from day 3 after culture are shown in **Figure 3B**.

### 3.4 Gene ontology analysis

Gene ontology (GO) analysis is a representative set of contigs in particular metabolic networks. GO classification considers three categories: molecular function, biological process and cellular component. The most substantial enrichments (*p* value < 0.05) in function and process are shown in **Figure 3C**.

### 3.5 Kyoto Encyclopaedia of Genes and Genomes (KEGG) analysis

The results of the KEGG database analysis are presented in **Supplementary Table 1**. There were significant changes in pathways related to *G. spinigerum* L3 infection. On day 1 after culture, the nucleotide-binding and oligomerisation domain-like receptor signalling pathway was significantly expressed, while on day 3, expression of the B cell receptor (BCR) signalling pathway and apoptosis pathway dominated.

### 3.6 Reactome analysis

**Figure 4:**
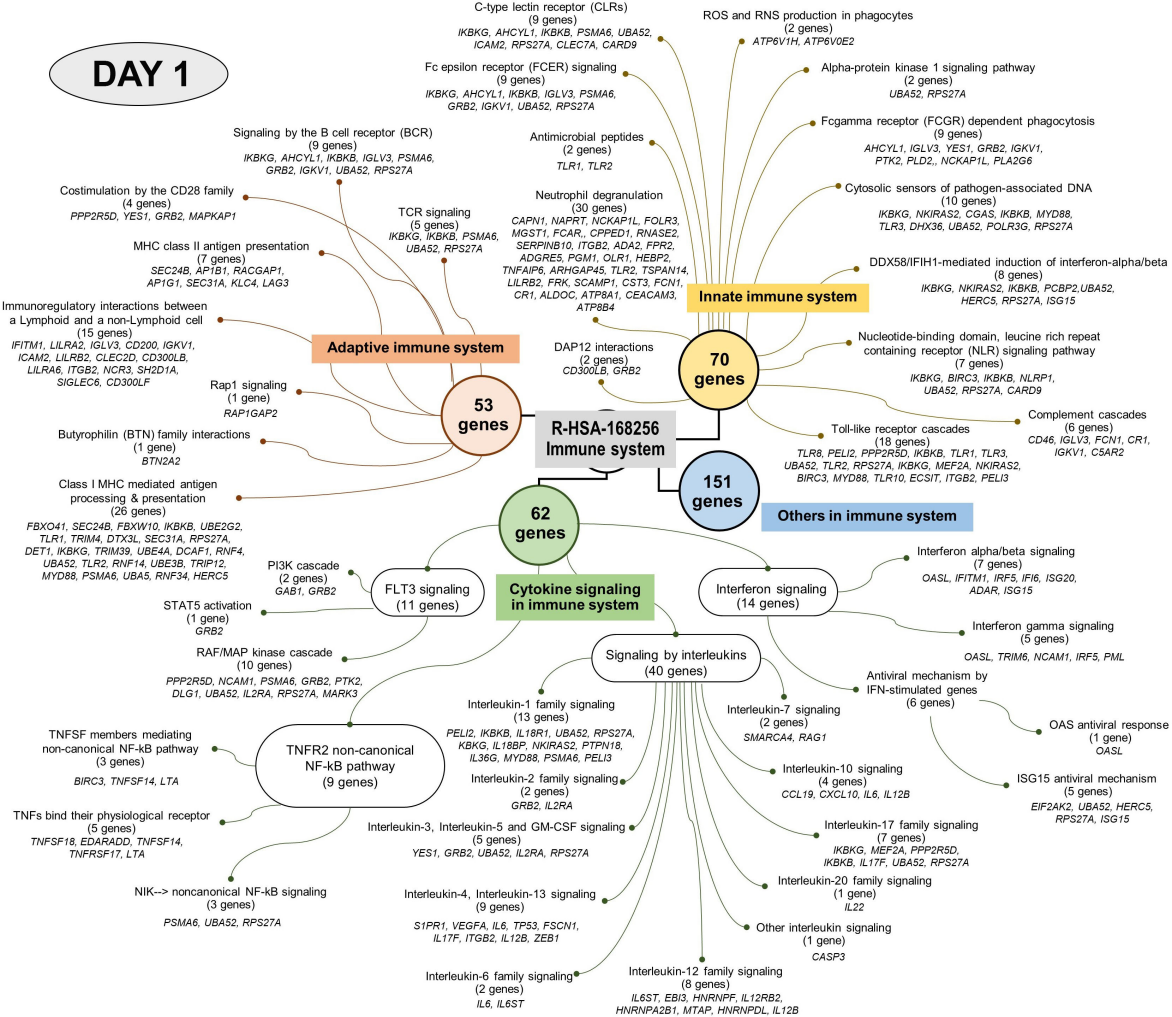
Visualised graph of immune system term (REAG : HAS-168256) of reactome database in PBMC co-cultured with *G. spinigerum* L3 at day 1.

**Figure 5:**
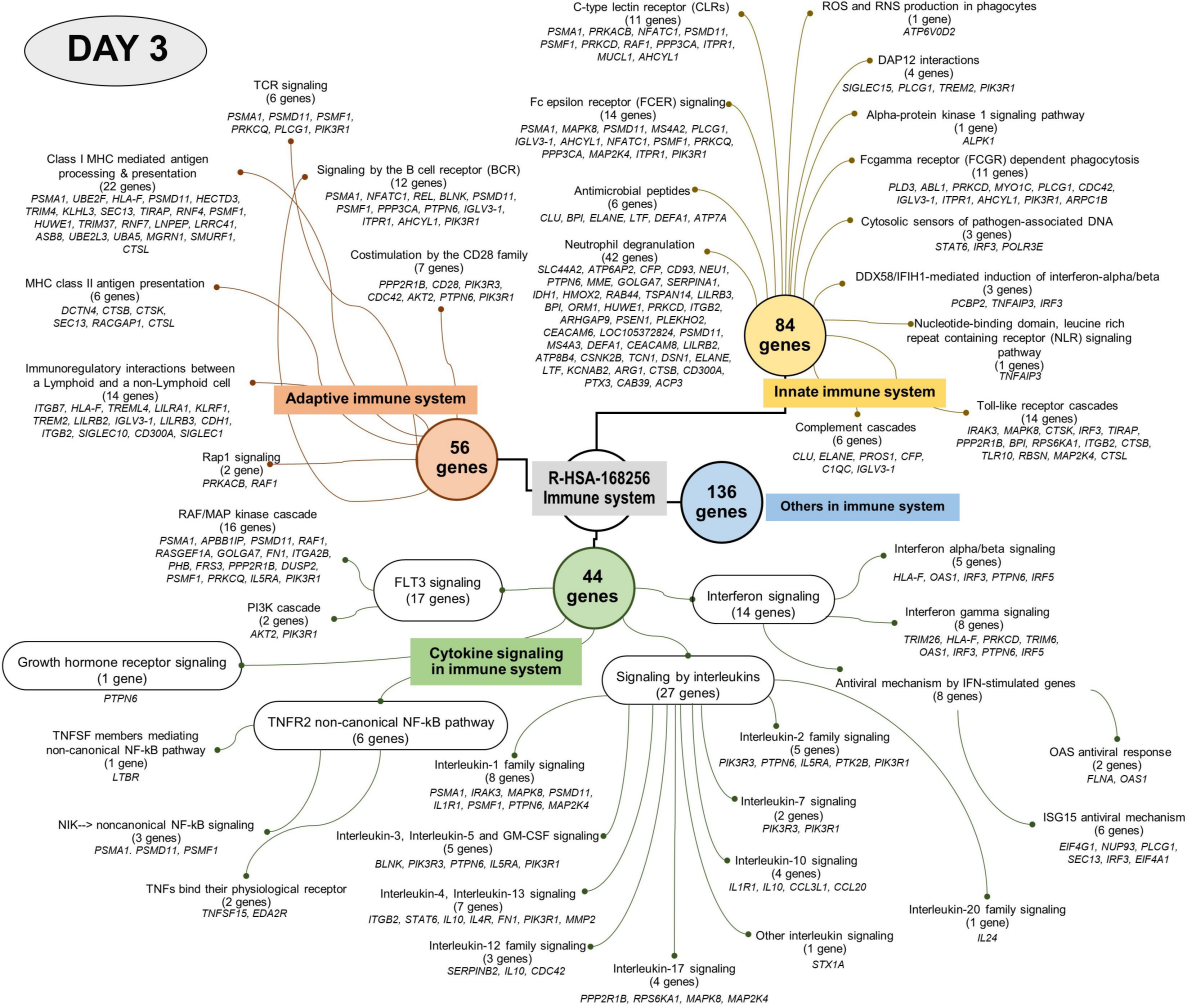
Visualised graph of immune system term (REAG : HAS-168256) of reactome database in PBMC co-cultured with *G. spinigerum* L3 at day 3.

**Figure 6:**
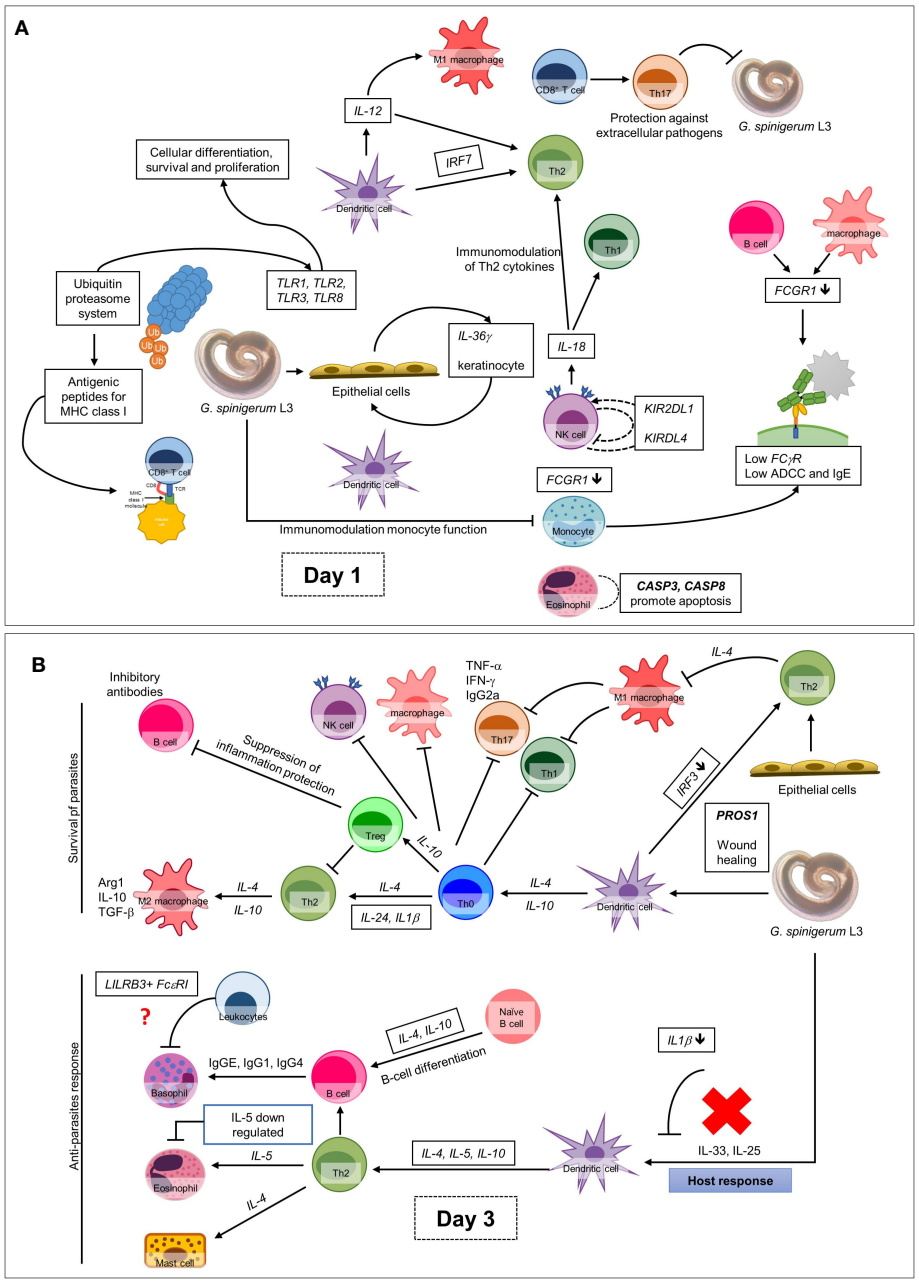
Overall up and down-regulated gene expression in PBMC co-cultured with *G. spinigerum* L3 **(A)** At day 1 after cultivation, gene expression profile trends to related to dominant innate immunity that play roles in pathogenesis, immunomodulation and contribute to evasion of *G*. *spinigerum* L3. The genes which play roles in pathogen recognition, proteolysis, NF-kB antigenic peptides generation for MHC I, NK cells, cytotoxic T cells activation, regulatory T cell, M1 macrophages, pro-inflammatory cytokines were up-regulated. Some of these can also drive type 2 immunity. Genes related to activation of NK cells, phagocytosis and complement system were down-regulated. **(B)** The genes expressed at day 3 of cultivation include genes related to regulation of T-cell activation and development, TCR and BCR which drive to Th 2 response, B cell development and antibody production, anti-inflammatory cytokines, wound healing and most of complement system were up-regulated. Genes play roles in phagocytosis function of monocyte, eosinophil, NK cell apoptosis, M2 macrophage and Th1/Th17 were down-regulated.

Analysis using the Reactome pathway databases revealed the significant expression of 18 pathways related to immune response on day 1 (**Figure 4**; **Supplementary Table 2**), but only seven pathways on day 3 after culture (**Figure 5**; **Supplementary Table 3**). Among the pathways identified as significant, the immune system pathway (REACT:R-HSA-168256) was significantly expressed on day 1 after culture, with the highest member count of 298 on day 1 and 276 counts on day 3 after culture. All non-human identifiers were converted to their human equivalent. The report was also filtered to only show results for the species ‘Homo sapiens’, and resources were set to ‘all resources’. The gene profiles from these data were grouped, and the association of those genes of interest that were involved in the immune response and were statistically significant on day 1 and day 3 after culture (*p* value < 0.05, FDR < 0.05) and related with gnathostomiasis caused by *G. spinigerum* L3 are shown in **Tables 1**, **2**, respectively. Proteins that showed significant changes in transcript levels on days 1 and 3 after culture were classified as involved in innate immunity, adaptive immunity, cytokine signalling in the immune system and others (**Figures 6A, B**).

**Table 1:** Significant regulated genes expression related to immune system in PBMC co-cultured with *G. spinigerum* L3 at day 1 of stimulation (*p* < 0.05 and FDR < 0.05).

Ensembl Transcript ID	Symbol	Description	logFC	*p*-value	FDR	Regulation
Toll like receptors						
ENST00000504367	*TLR3*	toll like receptor 3	11.19036612	2.41064E-05	0.000405919	up
ENST00000311912	*TLR8*	toll like receptor 8	4.990818373	0.002322798	0.016875361	up
ENST00000308979	*TLR1*	toll like receptor 1	4.824035063	0.003285048	0.022943438	up
ENST00000642580	*TLR2*	toll like receptor 2	-13.05858833	9.65844E-07	4.19988E-05	down
C-type lectins						
ENST00000438351	*CLEC5A*	C-type lectin domain containing 5A	4.546921524	0.005836801	0.037626389	up
ENST00000290855	*CLEC2D*	C-type lectin domain family 2 member D	4.041883774	0.008074238	0.049625056	up
Ubiquitine genes						
ENST00000461934	*UBA3*	ubiquitin like modifier activating enzyme 3	9.60663781	0.000358854	0.003131692	up
ENST00000450743	*UBE2D4*	ubiquitin conjugating enzyme E2 D4 (putative)	6.244955135	0.000535236	0.004449826	up
ENST00000449510	*UBE3B*	ubiquitin protein ligase E3B	4.246364235	0.007344852	0.045688205	up
ENST00000330942	*UBE2G2*	ubiquitin conjugating enzyme E2 G2	-5.625077179	0.001620904	0.012236419	down
ENST00000431736	*UBE4A*	ubiquitination factor E4A	-7.004217532	8.7854E-05	0.00108468	down
Proteasome						
ENST00000389993	*PSME4*	proteasome activator subunit 4	13.56332621	4.03346E-07	2.44619E-05	up
ENST00000559042	*PSME2*	proteasome activator subunit 2	5.396570406	0.001279515	0.009904009	up
ENST00000559741	*PSME1*	proteasome activator subunit 1	5.48867216	0.002764619	0.01896649	up
ENST00000592169	*PSME3*	proteasome activator subunit 3	-5.84535768	0.000459179	0.003754016	down
Caspase						
ENST00000490682	*CASP8*	caspase 8	5.024180794	0.001651213	0.012446583	up
ENST00000619992	*CASP2*	caspase 2	-11.56850313	1.26397E-05	0.000253086	down
ENST00000489932	*CARD9*	caspase recruitment domain family member 9	-9.872026654	0.000227724	0.002219461	down
IRF						
ENST00000528413	*IRF7*	interferon regulatory factor 7	4.128725299	0.007116144	0.044512773	up
LILRs						
ENST00000473156	*LILRA1*	leukocyte immunoglobulin like receptor A1	-14.96020652	3.59684E-08	6.66447E-06	down
ENST00000430421	*LILRA6*	leukocyte immunoglobulin like receptor A6	-10.95645976	3.60864E-05	0.00055212	down
Pro-inflammatory cytokines						
ENST00000259205	*IL36G*	interleukin 36 gamma	15.34778506	1.8383E-08	5.06752E-06	up
ENST00000648487	*IL12RB2*	interleukin 12 receptor subunit beta 2	11.25678998	2.14701E-05	0.000372734	up
ENST00000336123	*IL17F*	interleukin 17F	10.67675085	5.83948E-05	0.0007954	up
ENST00000538666	*IL22*	interleukin 22	9.784421306	0.000264562	0.002497361	up
ENST00000258743	*IL6*	interleukin 6	4.682488472	0.00287209	0.020372635	up
ENST00000379959	*IL2RA*	interleukin 2 receptor subunit alpha	4.160496605	0.006759739	0.042611299	up
ENST00000409599	*IL18R1*	interleukin 18 receptor 1	-12.16996757	4.47137E-06	0.000120186	down
ENST00000620017	*IL18BP*	interleukin 18 binding protein	-10.12432144	0.000149619	0.00160778	down
KIRs						
ENST00000336077	*KIR2DL1*	killer cell immunoglobulin like receptor, two Ig domains and long cytoplasmic tail 1	-12.809832	1.4849E-06	5.49861E-05	down
ENST00000463062	*KIR2DL4*	killer cell immunoglobulin like receptor, two Ig domains and long cytoplasmic tail 4	5.451300899	0.002834143	0.020140316	up
ENST00000479407	*NKIRAS2*	NFKB inhibitor interacting Ras like 2	-5.856937956	0.000910973	0.007277095	down
Complements components						
ENST00000472581	*CFB*	complement factor B	4.907049998	0.00322678	0.022580291	up
ENST00000595464	*C5AR2*	complement component 5a receptor 2	-12.36066091	3.22355E-06	9.53967E-05	down
ENST00000592860	*CFD*	complement factor D	-11.93525773	6.71648E-06	0.000159895	down
Surface proteins (CD)						
ENST00000473539	*CD200*	CD200 molecule	6.499255811	0.001050189	0.008284146	up
ENST00000528435	*CD3E*	CD3e molecule	4.415287189	0.007812412	0.048203957	up
ENST00000443761	*CD1C*	CD1c molecule	-5.272358835	0.002385781	0.017278145	down
ENST00000322875	*CD46*	CD46 molecule	-4.536967506	0.003832954	0.026264202	down
Others						
ENST00000433677	*CPPED1*	calcineurin like phosphoesterase domain containing 1	-13.39690662	5.3798E-07	2.89361E-05	down
ENST00000637481	*MEF2C*	myocyte enhancer factor 2C	-14.88729372	4.08259E-08	7.24487E-06	down

**Table 2:** Significant regulated genes expression related to immune system in PBMC co-cultured with *G. spinigerum* L3 at day 3 of stimulation (*p* < 0.05 and FDR < 0.05).

Ensembl Transcript ID	Symbol	Description	logFC	*p*-value	FDR	Regulation
Ubiquitine genes						
ENST00000346330	*UBE2A*	ubiquitin conjugating enzyme E2 A	9.447272478	0.000466677	0.003803408	up
ENST00000442670	*UBE2E1*	ubiquitin conjugating enzyme E2 E1	7.770827761	3.41012E-05	0.000517072	up
ENST00000414443	*UBE2F*	ubiquitin conjugating enzyme E2 F (putative)	6.335637296	0.000391682	0.003284291	up
ENST00000520595	*UBE2V2*	ubiquitin conjugating enzyme E2 V2	5.725884142	0.001874872	0.013466752	up
ENST00000545681	*UBE2L3*	ubiquitin conjugating enzyme E2 L3	4.277789464	0.007493316	0.044852236	up
ENST00000513098	*UBE2D3*	ubiquitin conjugating enzyme E2 D3	-6.097929538	0.000511692	0.004127887	down
Proteasome						
ENST00000559741	*PSME1*	proteasome activator subunit 1	5.48867216	0.002764619	0.01896649	up
ENST00000592169	*PSME3*	proteasome activator subunit 3	-5.84535768	0.000459179	0.003754016	down
Caspase						
ENST00000527979	*CASP1*	caspase 1	-12.28343431	3.67906E-06	0.000100176	down
IRF						
ENST00000597198	*IRF3*	interferon regulatory factor 3	-9.819519142	0.000247822	0.00230753	down
LILRs						
ENST00000391750	*LILRB3*	leukocyte immunoglobulin like receptor B3	12.63548282	2.00566E-06	6.59219E-05	up
ENST00000430952	*LILRB4*	leukocyte immunoglobulin like receptor B4	4.053443852	0.00790473	0.046871459	up
ENST00000391749	*LILRB2*	leukocyte immunoglobulin like receptor B2	-6.379914479	0.000168318	0.001717255	down
ENST00000251372	*LILRA1*	leukocyte immunoglobulin like receptor A1	-4.406010665	0.004825174	0.030807165	down
Anti- inflammatory cytokine						
ENST00000170630	*IL4R*	interleukin 4 receptor	5.154344032	0.001360231	0.010104634	up
ENST00000423557	*IL10*	interleukin 10	4.677601477	0.002936296	0.020020635	up
ENST00000391929	*IL24*	interleukin 24	4.567393135	0.004273952	0.02782438	up
ENST00000615950	*ILF2*	interleukin enhancer binding factor 2	-12.04247883	5.57705E-06	0.000135081	down
ENST00000416750	*IL1B*	interleukin 1 beta	-10.16892142	0.000137239	0.001470078	down
ENST00000383846	*IL5RA*	interleukin 5 receptor subunit alpha	-9.740896694	0.000282929	0.002562356	down
ENST00000472292	*IL1RN*	interleukin 1 receptor antagonist	-7.719125693	6.47064E-05	0.000834787	down
Complement complement						
ENST00000510260	*C1QB*	complement C1q B chain	10.6966174	5.6334E-05	0.000750665	up
ENST00000418949	*C2*	complement C2	8.521279704	3.54456E-05	0.000533091	up
ENST00000461983	*C1S*	complement C1s	5.525285908	0.00113524	0.008587219	up
ENST00000374640	*C1QC*	complement C1q C chain	5.044784209	0.001624009	0.011845263	up
Surface proteins						
ENST00000458610	*CD28*	CD28 molecule	4.820673844	0.002695362	0.018547355	up
ENST00000492627	*CD81*	CD81 molecule	4.724752731	0.003205828	0.021622473	up
ENST00000310828	*CD300A*	CD300a molecule	-6.445742921	0.000283966	0.002570811	down
ENST00000246006	*CD93*	CD93 molecule	-5.359648103	0.000934207	0.007186163	down
B-cell						
ENST00000413476	*BLNK*	B cell linker	9.66225397	0.00032541	0.00284507	up
Others						
ENST00000394236	*PROS1*	protein S	10.9802551	3.4689E-05	0.00052329	up

## 4 Discussion

Current knowledge about the pathogenesis, host-parasite interaction and immunity in human gnathostomiasis remains unclear.

In this study, the ultrastructural results revealed that EV-like particles were found both inside the *G. spinigerum* L3 body and the ESP pellet. The presence of EV-like vesicles in the parasite body was confirmed using transmission electron microscopy (TEM). In the present study, isolated EVs from *G. spinigerum* L3 were identified under TEM and presented the morphological characteristics as previously described (11, 12) We found that the vesicle sizes were consistent with exosomes and MVs reported in previous studies (11, 12), although the morphology and double membrane were not always obvious. This limitation may depend on the procedure used to prepare and process the samples for ultrastructural investigation. In a previous study, fresh *H. polygyrus* larvae were collected and transferred into a culture medium for only 5 h prior to staining for electron microscopy (12). In contrast, we examined a 3-day culture instead of fresh larvae. We suggest that the longer delay in examining the harvested larvae may have increased the degradation of the parasite organelles and cells. Additionally, most studies used ultracentrifugation for EV isolation and purification (12). Although this is the gold standard, high-speed centrifugation without preservation by sucrose gradient solution (24) can damage the EV membrane, as observed in the present study. However, alternative techniques, such as immunohistochemistry with antibody to EVs markers and molecular markers, are required to clarify our findings.

A previous study showed that 31 of 51 identified proteins in exosomes in *Schistosoma mansoni* (*S. mansoni*) ESPs (24). *S. japonicum-*derived EVs showed a high proportion of membrane- and tegument-associated proteins (25). The genetic and phenotypic profiles of EVs of several important pathogenic helminths have been reported (12). Here we did not perform any in-depth investigations to clarify and validate the functions and properties of the genome, transcriptome, and proteome in the EVs. However, this information is necessary to contribute to understanding the correlation of EVs and ESPs with the pathological effects, immunomodulation and immune evasion mechanisms of *G. spinigerum* L3.

The present study focused on exploring the gene expression related to both the innate and adaptive immune response against *G. spinigerum* L3 *in vitro* using PBMCs as an immune cell model. The human gene profiling results related to the immune response were obtained from NGS of PBMCs that were co-cultured with live *G. spinigerum* L3 for 3 days. Therefore, the PBMCs are representative of first-line immune cells in response to an infection. Additionally, PBMCs detect pathogen-associated molecular patterns (PAMPs) on the pathogen surfaces, transmitting signals into the cell to activate gene expression of pro-inflammatory and anti-inflammatory cytokines and chemokines (26–28). Therefore, PBMCs are influential biological sensors of infection, making them suitable for use as immune cell models for studying immune response against various antigen, for examples, studies in innate and adaptive immunity, immunoregulation, immunomodulation, and acute/chronic infection (29). However, sampling of mRNA levels obtained from PBMCs reflects a static point in time and may not illustrate the complete expression pattern (29). Similar to previous studies, we observed the expression of genes related to innate and adaptive immune responses at day 1 (30, 31) and day 3 after culture, respectively (32). Innate immune response can occur rapidly after innate immune cells are exposed to antigens. Previous studies in innate immunity, e.g. monocyte and NK cell activation, and pro-inflammatory cytokine secretion, observed the related gene expression at day 1 of stimulation. Adaptive immunity, such as T and B cell activation and antibody production, is noted about 7 days or later. Duration time to study adaptive immune gene expression is optimal from day 3 of incubation (33). So, in the present study we designed to investigate gene expression of innate and adaptive immunity at days 1 and 3 of stimulation. The previous study suggested that the PBMC expression profiles were drawn from a mixed population of cells and, therefore could not be attributed to a specific cell type or lineage (29). The composition of this profile reflects the conditions in the periphery and not those in germinal centres (GCs), lymph nodes or remote sites of infection and inflammation (29). In the present study, we selected genes of interest that were related to the immune response against *G. spinigerum* L3 on days 1 and 3 after culture and linked to conceivable pathogenesis, immunomodulation and immune evasion mechanisms in human gnathostomiasis.

**Table 1** summarises gene expression in human PBMCs against *G. spinigerum* L3 on day 1 after culture. The expression of several genes is related to immunity. Genes coding for toll-like receptors (TLRs), including *TLR1*, *TLR3* and *TLR8*, were mostly upregulated. TLRs are pathogen recognition receptors (PRRs). *TLR* upregulation drives T helper (Th) 2 or regulatory responses in *Acanthocheilonema viteae* infection (34) and schistosomiasis (35). TLRs also transduce signalling to promote the expression of genes coding for pro-inflammatory cytokines and type I interferons (IFNs; including IFN-α and IFN-β). They also promote innate immunity by activating the signalling transduction cascades for nuclear factor-κB (NF-κB), mitogen-activated protein kinases and interferon regulatory factor (IRF) 3 cascades (36). *TLR2* downregulation has been suggested to increase the ability to promote a Th2-type response (35). TLR2 is a key molecule required for innate immunity and is involved in the recognition of a wide range of viruses, bacteria, fungi and parasites. A cysticercosis experiment in a previous study demonstrated that the lack of Th1-dominant adaptive immunity in TLR2^−^/^−^ mice were associated with significantly elevated parasite burdens. While TLR2^+^/^+^ mice were resistant to infection (37). Furthermore, this Th2-type response is elicited by most helminth infections e.g. schistosomiasis, and stimulates the production of cytokines and interleukins (ILs), antibody isotypes (IgG1, IgG4 and IgE) (38) and expanded populations of eosinophils, basophils, mast cells, type 2 innate lymphoid cells and alternatively activated macrophages (M2) (39). In our previous study, human B cells separated from PBMCs were co-cultured with crude Ag derived from *G. spinigerum* L3 for 2 wks. The results revealed that cultured B cell function was impaired to produce specific IgM and IgG against the parasite antigens (19). The finding may contribute to *G. spinigerum* L3’s survival from the host’s immune response.

Earlier studies identified a family of membrane-bound TLRs (TLR1–TLR13), and mouse genetic studies revealed that TLRs generally serve as PRRs that recognise a wide range of PAMPs, including lipids, lipoproteins, proteins, glycans and nucleic acids and play a central role in initiating innate immune responses (40). In animal model studies, a loss-of-function mutation of the mouse homolog of hToll was subsequently unable to promote innate immune responses against bacterial lipopolysaccharides (41). Similarly, our present findings revealed *TLR2* downregulation, which may explain the defect in innate immunity in gnathostomiasis.

Similar to the expression of TLRs, CLR genes *CLEC5A* and *CLEC2D* code for C-type lectin, which served as PRRs were up-regulated on day 1 after culture. CLR proteins have a diverse range of functions, including cell-cell adhesion, immune response to pathogens and apoptosis (42). CLEC2D is a receptor for KLRB1, a natural killer (NK) cell receptor that protects target cells against NK cell-mediated lysis (43). CLEC5A, also known as myeloid DAP12-associating lectin 1(MDL-1), is highly expressed in myeloid lineages, such as neutrophils, monocytes and macrophages, as well as osteoclasts, microglia and dendritic cells (44). Both CLEC2 and CLEC5A are critical in microbe-induced “neutrophil extracellular trap” (NET) formation (a form of neutrophil activity to destroy pathogens) and proinflammatory cytokine production in viral infections (45). For example, dengue virus (DV) and H5N1 influenza A virus (IAV) could activate CLEC2 to induce the release of EVs, which further enhance damage in infected cells by NET (NETosis) and proinflammatory cytokine production *via* CLEC5A and TLR2 (45). In *Listeria monocytogenes* (*L. monocytogenes*) infection, CLEC5A induce NET formation and the production of proinflammatory cytokines and reactive oxygen species (ROS). Inoculation of *Clec5a* −/− mice with *L. monocytogenes* develops rapid bacterial spread, increased bacterial loads in the circulation and liver, and severe liver necrosis. In these knockout mice, IL-1β, IL-17A, and TNF production is suppressed (46). It is possible that *TLR* expression in the present study may have contributed to the ability of immune cells to recognise and eliminate parasites *via* the Th2 response. *TLR* and *NF-κB* expression protect against pathogenic infection by orchestrated gene expression programmes. Moreover, TLR ligands are potent activators of the NF-κB pathway that promote degradation of inhibitory IκBα, resulting in NF-κB activation (47). In the present study, *NKIRAS2*, which codes for NF-κB inhibitor interacting Ras-like 2, was downregulated, leading to NF-κB activation. NF-κB controls the transcription of cytokines and genes that regulate cellular differentiation, survival and proliferation. Additionally, it regulates the expression of various genes involved in innate and adaptive immune responses (48). Similarly, an in-vitro study in mouse macrophage co-cultured with ES from adult *Toxocara canis* revealed that NF-κB were expressed at both transcriptional and translational levels after 9 h of incubation. The production of pro-inflammatory cytokines, including TNF-α, IL-1β and IL-6 released by the stimulated macrophages, were modulated (49).

Ubiquitin (Ub) was originally named ATP-dependent proteolysis factor 1. Mammals have four Ub genes. *UBA52* and *RPS27A* code for Ub-ribosomal fusion proteins L40 and S27a, respectively, and *UBB* and *UBC* code for polyubiquitin precursor proteins (50). Ubiquitination refers to the conjugation of Ub to a target protein. This is a multi-step process that requires three enzymes: a Ub-activating enzyme (E1), a Ub-conjugating enzyme (E2) (51) and a Ub ligase (E3) (52). Ubiquitination is an energy-dependent, post-translational modification process and is suggested as essential for the initiation, maintenance and termination of the immune system’s response (50, 53). Ubiquitination affects cellular processes by regulating protein degradation *via* proteosomes (PSMEs) and lysosomes, coordinating the cellular localisation of proteins, activating and inactivating proteins and modulating protein-protein interactions. In response to the eradication of invading pathogens and to reduce concomitant host damage due to pathogen infection, the Ub system tunes the host’s innate immune system, including inflammatory signalling, phagosomal maturation, autophagy and apoptosis (53). Additionally, Ub mediates regulation of the TLR, retinoic acid-inducible gene-I-like receptor and tumour necrosis factor (TNF)-α signalling pathways. However, pathogens have evolved strategies to evade host innate immunity by usurping the Ub system to favour their own survival (54).

The PSME, a profoundly complicated protease complex, performs selective, efficient and processive hydrolysis of client proteins. PSME collaborates with Ub, which polymerises to form a marker for regulated proteolysis in eukaryotic cells (55). IFN-γ-inducible PSMEs or immunoproteasomes are a type of proteasome that degrades Ub-labelled proteins found in the cytoplasm under conditions of oxidative stress and in cells exposed to pro-inflammatory stimuli. Additionally, the immunoproteasome influences inflammatory disease pathogenesis through its ability to regulate T cell polarisation (55). The immunoproteasome contributes to the production of peptide epitopes for cytotoxic T cells and has been highlighted in the major histocompatibility complex (MHC) class I-restricted antigen-processing pathway and cell-mediated immunity (55, 56).

The degradation of ubiquitinated proteins is mediated by the Ub–proteasome system (UPS). Degradation of proteins by the UPS is the first step in the generation of MHC I-presented peptides (56). Protein degradation through the UPS is the major pathway of non-lysosomal proteolysis of intracellular proteins. It plays important roles in a variety of fundamental cellular processes, such as the regulation of cell cycle progression, division, development and differentiation; apoptosis; cell trafficking and modulation of the immune and inflammatory responses. Aberrations in this system lead to the dysregulation of cellular homeostasis and the development of various diseases (55, 57).

A recent transcriptomic and proteomic analysis in the immature stage liver fluke revealed that ubiquitin related genes were predominated. These genes regulate proteins at the cellular level *via* the ubiquitin proteasome system. It is specifically important for controlling cell cycle progression during intensified cell growth and proliferation in fascioliasis. In *Fasciola hepatica* infection, the major pathogenesis associated with results from the extensive tissue damage caused by immature fluke migration, growth and development in the liver. This is compounded by the pathology caused by host innate and adaptive immune responses against infection and repair tissue damage (58).

In this study, *UBA3*, *UBE2D4* and *UBE3B* were upregulated, while *UBE2G2* and *UBE4* were downregulated. Our findings indicated that Ub genes regulate functions by balancing the expression of Ub-activating, Ub-conjugating and Ub ligase enzymes. In agreement with our findings, Qureshi et al, suggested that alterations in the UPS would profoundly affect immune responses, including the regulation of an array of inflammatory cytokines. Additionally, the proteasome acts as a central regulator of inflammation and macrophage function *via* several pathways (59).

*PSME1, PSME2* and *PSME4* were significantly upregulated during our 3-day observation, while *PSME3* was significantly downregulated. Previous studies have suggested that IFN-γ-inducible PSME1 and PSME2 are components of the immunoproteasomes that play a crucial role in the generation of antigenic peptides for presentation on MHC I molecules and activation of the NF-κB pathway (55, 56, 59). Thus, our findings suggest that *G. spinigerum* L3-derived antigenic peptides were generated for MHC I presentation.

The IRFs are a family of transcription factors that play essential roles in various aspects of the immune response, including immune cell development and differentiation and regulating responses to pathogens. *IRF3, IRF5* and *IRF7* are vital to IFN-I production downstream of PRRs, and *IRF9* regulates IFN-driven gene expression. Additionally, *IRF4, IRF8* and *IRF5* regulate myeloid cell development and phenotype, thereby regulating inflammatory responses. IRF release during infection enhances the expression of IFN-I genes, IFN-stimulated genes and other pro-inflammatory cytokines/chemokines (60). Webb et al, reported that during *S. mansoni* infection, the parasite eggs stimulate plasmacytoid dendritic cells (pDCs) to potently induce IFN-I signalling, thereby driving Th2-type immunity, which is essential against helminthic infection (61). Acute *S. mansoni* infection and the upregulation of IFN-I enhance DCs, which activate T cell function (39, 61). *IRF7* upregulation was noted in the lymphoid tissue, and largely pDC activation (60). In the infection of lymphoid tissues, the majority of *IRF7* is expressed in pDC. Therefore, the *IRF7* upregulation observed in the present study suggests that increased IFN-I drives Th2-type immunity, leading to T cell activation against *G. spinigerum* L3 infection.

The leukocyte immunoglobulin-like receptors (LILRs) are in a family of immunoregulatory receptors comprising inhibitory (*LILRB 1–5*) and activating receptors (*LILRA 1–6*, excluding *LILRA 3*) (62, 63). (*LILRs* are expressed by haematopoietic cells, including monocytes, macrophages, dendritic cells, granulocytes, NK cells, T cells and B cells and non-immune cells such as endothelial cells and neurons (63). The inhibitory receptors contain an immunoreceptor tyrosine-based inhibitory motif, while the activating receptors couple with an immunoreceptor tyrosine-based activating motif ITAM)-bearing FcϵRI-γ (62). *LILRs* also modulate TLR signalling and functions. Thus, *LILRs* can modulate a broad set of immune functions, including immune cell function, cytokine release, antibody production, and antigen presentation. Additionally, LILRs in neutrophils activate and suppress antimicrobial responses. However, several human pathogens take advantage of these inhibitory receptors for immune evasion. In this study, downregulation of activating receptors, *LILAR1* and *LIAR6*, may contribute to *G. spinigerum* L3 evasion from host immune response.

Fc receptors for IgG (FcγRs) are broadly expressed by haematopoietic cells and consist of one inhibitory and several activating receptors that differ in their affinity and specificity for immunoglobulin subclasses (64). Our earlier study suggested that the level of FcγRI (CD 64) expression on monocytes was depleted by *G. spinigerum* L3 ESPs, leading to impaired phagocytic function (30). This agreed with our finding of the downregulation of *FCGR1*, which codes for FcγRI, during the first 24 hours of the co-culture of *G. spinigerum* L3 with PBMCs. FcγRI is an activating receptor with a high affinity for IgG and is expressed on monocytic DCs and monocytes/macrophages. The cross-linking of FcγRI (also called CD64) and antigen-antibody complex initiates signal transduction cascades for phagocytosis, cytokine production and antibody-dependent cell-mediated cytotoxicity (ADCC) (65). ADCC is dependent on eosinophils, neutrophils, macrophages or platelets as effector cells and IgE, IgG or IgA as antibodies. It also immobilises nematode larval stages as they migrate through the gut mucosa (66). In the present study, *FCGR1* downregulation suggests that the larvae modulate host immunity by impairing monocyte capacity and ADCC mechanisms. However, our findings suggest less efficiency in disrupting *G. spinigerum* L3 migration in human gnathostomiasis. Furthermore, *in vitro* co-culture of *H. polygyrus* larvae and bone marrow (BM)-derived macrophages found that the larval immobilisation was largely independent of CD11b and, instead, required the activating IgG receptor FcγRI. FcγRI signalling also contributed to the upregulation of macrophage Arg1 expression. IgG2a/c was the major IgG subtype in early immune sera bound by FcγRI on the macrophage surface (66). In addition, purified IgG2c and Arg1 expressed by macrophages elicited larval immobilisation (67).

Killer cell immunoglobulin-like receptors (*KIRs*) are expressed on NK cells and subsets of CD8^+^ T cells. KIRs inhibit the ability of cytotoxic cells to lyse cells with self-expressed MHC I alleles and are key regulators of the development, tolerance and activation of NK cells. KIR2DL4, composed of two Ig domains and long cytoplasmic tail 4, is a unique long-tailed activating KIR, and KIR2DL4 is only expressed on CD56 high NK cells. *KIR2DL4* expression results in a more potent activator of cytokine production rather than cytotoxicity and is associated with the ITAM-bearing FcϵRI-γ adaptor and LILRs. There are multiple similarities between KIRs and LILRs in terms of Ig domain-based structure, gene location and the ability to recognise MHC I (68). The present study revealed that *KIR2DL4* was upregulated, while the inhibitor receptor KIR2DL1, composed of two Ig domains and long cytoplasmic tail 1, was downregulated. KIR inhibitory receptors recognise self-MHC I molecules on target cells and consequently activate signalling pathways to prevent the cytolytic function of NK cells (68). However, KIRs that recognise the same MHC I molecule are usually not expressed by the same NK cell. Our observation of up- and downregulation to both inhibit and activate KIR balance suggests the modulation of NK cell capacity in natural infections of *G. spinigerum* L3. Moreover, our recent study in PBMCs exposed to ES from *G. spinigerum* L3 during the 7-day observation revealed low amounts of ES modulated NK cells by decreasing *IFNG* mRNA expression and IFN-γ production, and upregulating the expression of *NKG2A* and *NKG2D* encoded for C type killer cell lectin-like receptor (KLR), inhibitory receptor (NKG2A), and activating receptor (NKG2D). KLRs require some adaptors to initiate signalling transduction and cellular activation in NK cell cytotoxicity (43). Consistently, mice infected with *Echinococcus multilocularis* (*E. multilocularis*) larvae showed decreased NK cell frequencies and increased NKG2A expression on NK cells. These changes in NK cells during alveolar echinococcosis resulted in low cytotoxic activity through decreased IFN-γ secretion (69).

In the current study, *CD3E*, which controls cell surface proteins, was upregulated. CD3E proteins form the T cell receptor-CD3 complex, which plays an important role in coupling antigen recognition to several intracellular signal transduction pathways. It is also essential for T cell development (70). We found that *CD1C* and *CD46* were downregulated. Earlier studies suggested that CD1C molecules survey for lipid antigens throughout the endocytic system (71, 72). *CD46* codes for the complement regulatory protein C46. This is a cofactor of the CD3 T cell receptor, which is a receptor for complement components C3b and C4b. CD46 interferes with the inactivation and cleavage of C3b and C4b *via* serum factor I. CD46 protects the host cell from damage by complement *via* membrane-binding proteins and opsonisation (73). Therefore, the downregulated *CD46* expression observed in this study suggests that host tissue damage occurs due to complement activation. A previous study showed that helminth lipids, such as schistosome phosphatidylserine (PS), are also implicated in immune modulation. The schistosome PS induces DCs to polarise IL-4-/IL-10-producing T cells (74). It appears consistently that these expressions of gene-controlled cell surface proteins in this study may promote antigen recognition and T cell development.

The complement system is an effective host defence against initial infection by opsonisation, killing pathogenic organisms and recruiting inflammatory cells. The system is composed of three pathways of complement activation: the classical pathway, mannose-binding lectin pathway and alternative pathway (AP). Among the three pathways, the AP accounts for most of the complement activation. Complement factor B (FB), coded by *CFB*, is a major protease of the AP and circulates in the blood as a single-chain polypeptide. Upon activation of the AP pathway, FB is cleaved by complement factor D (FD), yielding the noncatalytic chain Ba and the catalytic subunit Bb. The active subunit Bb is a serine protease that associates with C3b to form the AP C3 convertase and proceed to the next step of parasite killing (75). Bb is involved in the proliferation of pre-activated B lymphocytes, while Ba inhibits their proliferation. *CFB* is localised in the MHC III region on chromosome 6 (76). In the present study, *CFB* was upregulated on day 1 after culture, while *CFD*, which codes for FD, was downregulated. These observations may be due to the low efficiency of FB cleavage, leading to impaired activation of the AP on day 1. Our finding agrees with a previous study. TsPmy is a paramyosin secreted by *Trichinella spiralis* (*T. spiralis*) on the surface of larvae and adult worms. Only P2 peptide of 9 peptides covering TsPmy241-280aa could interact with complement components C1q and C8/C9 to compromise their activation and functions. The binding of P2 peptide to C1q significantly inhibited both C1q-initiated complement classical activation and C1q-induced macrophage chemotaxis. This finding contributes to the ability of *T. spiralis* to evade host immunity (77).

Caspases are a class of cysteinyl proteases that play key roles in programmed cell death and inflammation. The human caspase gene family contains 11 members that are classified into three groups. Group 1 (*CASP1, CASP4* and *CASP5*) comprises enzymes involved in inflammation regulation. Group 2 (*CASP2, CASP3* and *CASP7*) and group 3 (*CASP6, CASP8, CASP9* and *CASP10*) comprise caspases that regulate apoptosis. Caspases play a central role in the execution phase of cell apoptosis. CASP8 is essential for death receptor-induced apoptosis (78) and initiates the extrinsic pathway, while CASP9 initiates the intrinsic apoptosis pathway, which is activated by dimerization induced when the activation and recruitment domain (CARD) of CASP9 binds to the adapter protein apoptotic protease-activating factor-1 (79). Active CASP9 then initiates apoptosis by cleaving and thereby activating executioner caspases. In this study, the initiator caspase (*CASP8*) and the executioner caspase (*CASP3*) were upregulated, while *CASP2* and *CARD9* were downregulated on day 1 after culture. These findings suggest that *G. spinigerum* L3-induced apoptosis of PBMCs occurs *via* extrinsic rather than intrinsic pathways. Supporting this, our previous study reported that *G. spinigerum* L3 ESPs also induced apoptosis of PBMC *via* the extrinsic pathway during a 48 hour-observation (80). Early apoptosis in ESP-induced PBMC was noted within 90 min post-exposure, and the greatest effects were found at 18-24 h. The regulatory genes involved in the apoptotic processes were expressed, including several caspases, especially *CASP3, CASP8*, and *CASP9*. In addition, the expression of 7 genes associated with extrinsic apoptotic pathways were increased, including *DAPL1, FADD, FAS, TNFRSF9, TNFSF8*, and *XIAP* (80). Similarly, *Paragonimus westermani* ESP products induced CASP3-mediated apoptosis of human eosinophils (81).

ILs are cytokines produced by leukocytes and several other body cells. They regulate cell growth, differentiation and motility and are particularly important in stimulating immune responses like inflammation (82). In this study, most genes that controlled pro-inflammatory cytokines, such as *IL-6, IL-12, IL-17, IL-22* and *IL-36*, were upregulated on day 1 after culture. In sepsis, the upregulation of these ILs probably activated or modulated the activity of various immune cells, such as IL-12-enhanced Th1 differentiation, NK cell activation, and classical macrophage (M1) activation. IL-17 prevents parasite evasion, IL-6 modifies regulatory T cells (Tregs), IL-22 is involved in parasite expulsion and IL-36 regulates keratinocytes in wound healing (82). In contrast, IL-18 was downregulated on day 3 after culture. IL-18 stimulation is mediated by IL-18 receptors. The binding of IL-18 to these receptors relays signals from myeloid differentiation primary response protein 88, a primary adapter protein for many TLR and IL-1R family members. IL-12 or IL-2 enhance the effect of IL-18 in immune cell activation. Together with IL-12, IL-18 promotes IFN-γ production from Th1 and B cells. Meanwhile, IL-18 alone is sufficient to induce the production of IFN-γ by NK cells. IL-18 not only induces Th1 cytokine production but also activates the humoral immune response *via* Th2 cytokine production (82, 83). Therefore, IL-18 downregulation may interfere with the complex interplay between cytokines related to IL-18 in the host cell-mediated or humoral immune response.

In schistosomiasis, biomolecules secreted by skin-penetrating cercariae, migrating schistosomulae, larval and adult worms, and their eggs, modulate both innate and adaptive immune responses. The overall interactions include the down- or up-regulated cytokines that activate or inhibit inflammation, and switches between the Th1 and Th2 immune response. Once infected with skin-penetrating cercariae, the initial response (for about 6 weeks) within infected tissues and plasma is characterised by type-1 inflammation, which is driven by IL-1, IL-12, TNF-α and IFN-γ. Subsequently, chronic schistosomiasis is developed mainly by the Th2 response to soluble antigens secreted by the eggs, and is driven by IL-4, IL-5, IL-10, and IL-13. IL-10 is an important element in disease progression by inducing inflammation through down-regulation of the Th1 response, while also preventing severe disease during the Th2 response (36).

In the present study, genes related to the nervous system were upregulated. *PSEN1* encodes the protein presenilin 1, which is described as the proteolytic subunit of γ-secretase (84). The γ-secretase complex is involved in processing amyloid precursor protein (APP). APP is manufactured in the brain and other tissues and is involved in the formation of neurons in the brain both before and after birth as well as in normal immune system function. β-amyloid (Aβ) is derived from β-APP through sequential cleavage by β- and γ-secretases. One of the most critical pathological features of Alzheimer disease is the accumulation of Aβ peptides that form extracellular senile plaques in the brain (84). In this study, the upregulated *PSEN1* may contribute to damage or degeneration during parasite migration in nerve tissues. However, further in-depth studies are required.

**Table 2** summarises gene expression in human PBMCs against L3 on day 3 of culture. The expression of several genes is related to immunity. E2s play a role in Ub size by twisting and attaching Ub to cellular proteins. Humans have approximately 40 E2s that are involved in Ub or Ub-like protein transfer (51). In the present study, most of the genes encoding E2s, including *UBE2A*, *UBE2E1*, *UBE2F*, *UBE2V2* and *UBE2L3*, were significantly upregulated on day 3 of culture. This suggests that the attachment of Ub to cellular protein may be increased. The expression of *UB* genes on both days 1 and 3 resulted in the movement of important proteins in the cells. On day 1 of culture, Ub was involved in the synthesis of new proteins for host defence, such as TLRs. Additionally, defective proteins are destroyed by ubiquitination.

On day 3 of culture, *PSME1* was upregulated, while *PSME3* was downregulated. Only PSME3 can promote NF-κB activity (56). Therefore, NF-κB activation may decline on day 3. Although Ub-related genes were highly upregulated, the expression level of *PSMEs* was low. The imbalance between the expression of *UB* and *PSME* genes may result in impaired protein degradation. In contrast to *PSME* expression on day 1 after culture, the lack of IFN-γ-inducible PSME2 may lead to incompletely formed immunoproteasomes. Additionally, hyperactivation of *IRF3* (85) was downregulated, leading to low expression of type I IFNs. These findings on day 3 possibly induced the decrease in MHC I antigenic peptide generation, which may lead to increased susceptibility to *G. spinigerum* L3 infection.

*LILRB3* and *LILRB4* were predominantly upregulated, and *LILRA1* was downregulated. The function of these ligands is unknown. The LILRB subfamily is considered immune checkpoint receptors that are crucial for maintaining self-tolerance and modulating the length and magnitude of physiological immune responses in peripheral tissues (86, 87). Lu et al, demonstrated that LILRB4 inhibited TNF-α release *via* FcγRI signalling and also inhibited inflammatory cytokine production (87). *CASP1*, which plays a major role in cytokine maturation (88), was downregulated. These findings support the modulation of cytokine involvement on day 3 of culture. Cytokines are important components of the immune system. They modulate the balance between humoral and cell-mediated immunity. Furthermore, they regulate the maturation, growth and responsiveness of immune cell populations. *BLNK*, which controls B cell development and B cell linker protein (89), was upregulated on day 3 of culture.

On day 3 after culture, *IL-4R*, *IL-10* and *IL-24* expression of cytokines was upregulated. Several studies have suggested that Th2 function is activated by IL-4, IL-24 and IL-1β (82). B cell differentiation and IgE production are activated by IL-4 and IL-10 (82). Furthermore, IL-10 production promotes the downregulation of Th1 cells, NK cells and macrophages and suppresses Th1/Th17 during helminthic infection (90). Additionally, IL-4 expression enhances MHC II expression. In contrast, the downregulation of *IL1B* observed on day 3 probably results in IL-25 and IL-33 suppression, supporting chronic helminthic infection. Moreover, IL-5 acts as an eosinophil chemoattractant factor. For example, in early infection, *S. mansoni* eggs induce IL-5 function in recruiting eosinophils to the site of antigen deposition (91, 92).

The TNF-encoding genes *TNFSF15*, *TNFAIP3* and *TNFRSF1B* were downregulated on day 3 after culture. TNF is used by the immune system for cell signalling and can induce fever, apoptotic cell death and inflammation (90). TNF-α functions through the regulation of IL-4 and IL-13 receptor expression, and it was reported to regulate Th2 cytokine responses that have protective immunity against *Trichuris muris* and *T. spiralis* infections (90). In the present study, the downregulation of *TNF* on day 3 of culture may be immunomodulation caused by the parasite, enabling survival in host cells.

Complement activation was predominant on day 3. *C2*, *C1s*, *C1QB* and *C1QC* were upregulated. Consequently, the elimination of the invading parasites may occur through the formation of a membrane attack complex *via* the classical pathway and promoting inflammatory reactions on the parasite’s surface. It has also been reported that several parasites secrete calreticulin, a Ca^2+^-binding chaperone protein. This protein mainly resides in the endoplasmic reticulum but is also found in other cellular compartments, including the plasma membrane. Some studies have demonstrated that calreticulin from helminth infections, such as *T. spiralis* (93), *Necator americanus* (94) and *E. multilocularis* (95), bind to C1q to interfere with the activation of the classical complement pathway. This phenomenon is one of the immune evasion strategies of these helminths, but there is no report of this in human gnathostomiasis. The present study found that genes related to the AP were downregulated on day 1. This suggests that the efficiency of the AP to kill parasites was decreased. Thus, our findings assumed that the classical complement pathway played a dominant role in the immune response during our 3-day observation.

On day 3 after culture, we found that genes regulating wound healing, particularly *PROS1*, which encodes protein S, were upregulated. Protein S is important for controlling blood clotting and a cofactor for activated protein C, which helps to prevent coagulation and stimulates fibrinolysis in the wound healing process (96). In contrast, the knockdown of *PROS1* in human glioma cells (LN18) caused the activation of both extrinsic and intrinsic apoptotic pathways and the inhibition of migration and invasion. This finding contributes to an important role in the development of glioblastoma multiforme, which is an aggressive brain tumour with poor prognosis (97). This knowledge may explain that up-regulated *PROS1* expression in the present study supports the migration and invasion of *G. spinigerum* L3

*CD28* and *CD81* were upregulated on day 3 of culture. CD81 is a tetraspanin protein, which is a type of protein consistently found within exosomes. CD81 has been identified as a marker for EVs and exosomes (98, 99). It is widely expressed on immune cells, such as B cells, T cells, NK cells, monocytes and eosinophils. Some studies have suggested that *CD81* shows the highest expression levels on GC B cells. On B cells, CD81 exists in a complex with CD19, CD21 and Leu13. CD81 plays a role in segregating the CD19/CD21–BCR complexes to lipid rafts to activate signal transduction. It is also involved in antigen presentation, T cell signalling, activation, motility and adhesion (100) and BCR signalling, B cell development and lymphocyte proliferation (101).

The expression of CD28 on T cells provides co-stimulatory signals required for T cell activation and survival (102). Furthermore, *CD28*−/− mice consistently showed increased susceptibility to *S. mansoni* infection by impaired Th2 response and also had reduced levels of immunoglobulin secretion (103).

Our findings revealed the downregulation of *CD93* and *CD300A*. CD93 is a receptor implicated in cell adhesion and cytoskeletal organisation and is expressed on developing B cells in the BM and on plasma cells but not on naive B cells, B cells in GCs and memory B cells (104). CD93 contributes to the long life of BM plasma cells (105). CD93 is also strongly expressed in platelets, megakaryocytes, endothelial cells, NK cells and monocytes. It is a C1q receptor of phagocytosis involving apoptotic bodies but does not bind directly to C1q (106). CD93 plays a role in cell adhesion, proliferation and migration, as well as in the regulation of inflammatory responses. Under inflammatory conditions, CD93 is proteolytically cleaved from the cell surface of myeloid cells and, as a soluble protein, is involved in opsonising apoptotic cells for efferocytosis by mouse and human macrophages. Efferocytosis is the effective clearance of apoptotic cells by professional and nonprofessional phagocytes. The process is mechanically different from other forms of phagocytosis and involves the localisation, binding, internalisation and degradation of apoptotic cells. Furthermore, it is important for inflammation resolution and homeostasis restoration (107). In mice, CD93 plays a role in the control of innate and adaptive immunity in the CNS (108). In the present study, we supposed that phagocytosis by monocytes and macrophages and apoptosis by NK cells might decrease due to the downregulation of *CD93* expression. Similarly, the uptake of apoptotic cells was impaired in mice with deficient CD93 expression (109).

CD300a is a member of the CD300 glycoprotein receptor family of cell surface proteins found on leukocytes, including monocytes, DCs, NK cells, neutrophils, basophils and T cells. Unlike CD93, CD300a acts as an inhibitory receptor and is involved in the diverse signalling pathways of both innate and adaptive immune cells. CD300a controls processes such as cellular differentiation and viability, cytokine/chemokine secretion, phagocytosis, inflammation and chemotaxis (110). The role of the CD300a receptor during *Leishmania* infection has been reported. The parasite induces CD300a receptors on the host’s phagocytic cells and antigen-presenting cells to counter the host’s defence mechanisms, which eventually facilitates parasite survival. The blocking of CD300a signals in *Leishmania* antigen-activated macrophages and dendritic cells enhances the production of nitric oxide and pro-inflammatory cytokines along with MHCI/II gene expression and reduces parasitic uptake (111). The inhibition of CD300a signals in mice infected with *Leishmania* also induced antigen-experienced cells (i.e. CD4^+^CD44^+^ and CD8^+^CD44^+^ T cells) to produce more pro-inflammatory cytokines and was helpful in the early clearance of parasites from visceral organs (111).

The present study had some limitations (1): we did not confirm EVs by immunostaining with antibodies to their markers (2); we did not identify EV components and validate their roles; and (3) we did not select important genes to verify whether they correlated with the pathogenesis of human gnathostomiasis. The pathogenesis of the disease is complex, resulting from the host’s immune response and the mechanisms of several larval factors to evade host attack. Further studies are strongly needed to clarify the effects of the larvae and their various biomolecules on host immunity, particularly type 1 and type 2 immunity, cytokine profiles, the network interplay of cytokines, immune cells and effector cells, and the mechanisms of the complement system and signalling pathways.

## 5 Conclusion

First, *G. spinigerum* L3 produce EVs and release them into the ESPs. However, the present study did not assess its components. Thus, further studies are necessary to identify the components and verify their roles in the pathogenesis and immunomodulation of human gnathostomiasis.

Second, human gene profiling of the immune response to the live larvae for 3 days revealed that the gene expression profile on day 1 of culture appeared to involve pathogenesis, immunomodulation and evasion. This includes the upregulation of genes that play roles in pathogen recognition, proteolysis, NF-κB antigenic peptide generation for MHC I, NK cells, cytotoxic T cell activation, Treg cell activation, regulatory T cells, M1 macrophages and pro-inflammatory cytokines, some which also drive type 2 immunity. However, genes related to innate immunity, particularly NK cells, phagocytosis and complement activation, were downregulated. On day 3 after culture, the genes that regulate T cell activation and development, T cell receptors and BCRs, which drive the Th2 response, gene-regulated B cell development, antibody production, anti-inflammatory cytokines, most of the complement systems and wound healing, were upregulated. In contrast, the phagocytosis function of monocytes, eosinophils, NK cell apoptosis, M2 macrophages and Th1/Th17 were downregulated. These findings may explain the immune evasion mechanisms of the larvae and the expression of some genes involved in adaptive immunity and others related to antibody production. Additionally, the results showed that most signalling from T and B cells drives Th2 immunity related to chronic infection and its pathogenesis.

Collectively, the findings of our investigation represent current knowledge, so that further in-depth studies are necessary to clarify how *G. spinigerum* L3 modulate the human immune response and the pathogenesis of gnathostomiasis.

## Data availability statement

The datasets presented in this study can be found in online repositories. The names of the repository/repositories and accession number(s) can be found below: https://www.ncbi.nlm.nih.gov/, PRJNA853915.

## Ethics statement

The studies involving human participants were reviewed and approved by the Ethics Committees of the Faculty of Tropical Medicine, Mahidol University. Written informed consent for participation was not required for this study in accordance with the national legislation and the institutional requirements. The animal study was reviewed and approved by Faculty of Tropical Medicine- Animal care and use committee.

## Author contributions

PP and WD were responsible for laboratory work including blood collection and culture experiments. TY collected and cultured third-stage *G. spinigerum* larvae. SB conducted RNA extraction and determined quality of RNA. PP, OR, and UB analysed the NGS data. WD worked on data analysis and statistical calculations. YM, PD, OR, and UB participated in the study design, conceived the study, and coordination. PP, WD, and YM wrote the manuscript. All authors contributed to the article and approved the submitted version.
