# Supplementary material for: Regulation of immune response against third-stage Gnathostoma spinigerum larvae by human genes
Source: Front Immunol. 2023 Aug 3;14:1218965. doi: 10.3389/fimmu.2023.1218965 (PMC10436992; doi:10.3389/fimmu.2023.1218965)
Supplement: Supplementary file 1 [file Table_1.docx]

**Supplementary Table 1**. Kyoto Encyclopedia of Genes and Genomes (KEGG).

The results show the significant changes of pathways related to *G. spinigerum* L3 infection. At day 1, NOD-like receptor signaling pathway was significantly expressed, while B cell receptor signaling pathway and apoptosis were dominated at day 3 of culture.

| **KEGG ID** | **KEGG term** | **Count** | ***p*-value** | **Genes** |
| --- | --- | --- | --- | --- |
| **Day 1** | | | | |
| KEGG:05168 | Herpes simplex virus 1 infection | 96 | 2.29E-08 | *ZNF43, ZNF7, ZSCAN32, IL12B, SRPK1, ZNF426, OAS1, ZNF773, ZNF778, TLR3, ZNF772, ZNF765-ZNF761, BIRC3, ZNF793, ZNF707, ZNF790, ZNF12, ZFP57, ZNF549, ZNF155, ITGA5, ZNF589, LTA, ZNF382, ZNF45, ZNF544, ZNF630, ZNF610, ZNF3, ZNF442, ZNF530, TP53, ZNF567, ZNF619, ZNF613, ZNF682, ZNF112, ZNF419, PIK3CD, ZNF470, ZNF415, ZNF845, SP100, TSC2, BAK1, ZNF37A, ZNF383, HLA-DMB, CGAS, ZNF180, ZNF268, ZNF195, CASP8, HLA-DQB1, IL6, TBK1, STAT2, ZNF226, ZNF107, IKBKB, TNFSF14, ZNF616, IFIH1, PML, ZNF875, ZNF600, EIF2AK2, OAS3, IRF7, CASP3, IFNGR2, ZNF570, ZNF460, ZNF84, MYD88, ZNF273, IKBKG, HLA-C, STING1, ZNF267, ZFP90, ZNF256, HLA-DPB1, CARD9*  *ZNF641, FAS, ZNF74, ZNF595, ZNF133, ZNF439, ZNF182, ZNF780B, ZNF627, ZNF254, TLR2, ZNF331* |
| KEGG:04621 | NOD-like receptor signaling pathway | 35 | 0.00425332 | *MFN1, OAS1, BIRC3, NOD2, NLRP7, TRPM2, ITPR1, P2RX7, CARD8, XIAP, RNF31, VDAC1, CASP8, IL6, TBK1, STAT2, GBP5, IKBKB, GBP3, OAS3, GBP1, IRF7,*  *GSDMD, PLCB1, CYBA, MYD88, IKBKG, STING1, NLRP1, CARD9, TP53BP1, ATG5, PSTPIP1, NLRP12, GABARAP* |
| **Day 3** | | | | |
| KEGG:05135 | Yersinia infection | 31 | 5.7787E-05 | *PXN, PIK3CA, PIK3R3, ACTR3B, AKT2, CCL2, DOCK1,*  *RPS6KA1, IL10, ACTR3C, PTK2B, NFATC1, IRAK4, NFATC3, PKN2, PIK3R1, RAC1, RELA, GIT2, MAPK8,*  *IRF3, FN1, IL1B, WIPF2, NFKBIA, MAP2K4, PIP5K1A,*  *ARHGEF1, CASP1, PLCG1, CDC42* |
| KEGG:04662 | B cell receptor signaling pathway | 23 | 0.00158643 | *LILRB3, PIK3CA, PIK3R3, BCL10, AKT2, BLNK, HRAS, RAF1, CD81, LILRB1, SOS1, PTPN6, LILRB4, LILRA1, NFATC1, DAPP1, NFATC3, PPP3CA, LILRB2, PIK3R1, RAC1, RELA, NFKBIA* |
| KEGG:05168 | Herpes simplex virus 1 infection | 77 | 0.00227789 | *ZNF585A, ZNF585B, OAS2, ZNF180, ZNF707, ZNF765, ZNF84, ZFP69, ZNF548, PIK3CA, ZNF155, ZNF570, ZNF7, ZNF343, PIK3R3, ZFP30, LTA, AKT2, ZNF547, ZNF248, ZNF568, ZNF436, TSC2, ZNF250, ZNF823,*  *ZNF530, ZNF205, ZNF211, FAS, ZNF468, CCL2, ZNF777, ZNF620, ZNF132, ZNF200, SRSF7, CFP, HLA-DPB1, HLA-F, ZNF184, ZNF8, ZNF836, ZNF621, IRAK4, SRSF3, BID, ZNF17, PIK3R1, OAS1, RELA, EIF2B5, ZNF544, ZNF169, IRF3, ZNF225, IL1B, ZNF268, ZNF793, ZNF619, ZNF615, ZNF773, NFKBIA, ZNF44, ZNF879, ZNF23, ZNF91, ZFP90, ZNF347, ZNF227, ZNF253, ZNF813, ZNF302, ZNF569, ZNF304, ZNF595, IFNGR2, ZNF41* |
| KEGG:05210 | Colorectal cancer | 22 | 0.00472448 | *PIK3CA, GADD45A, MYC, PIK3R3, MSH6, AKT2, DDB2, HRAS, MLH1, BBC3, APC, RAF1, SOS1, BRAF, EREG, PIK3R1, RAC1, MAPK8, TCF7, TCF7L2, BCL2L11, RPS6KB2* |
| KEGG:04380 | Osteoclast differentiation | 25 | 0.0105696 | *LILRB3, OSCAR, PIK3CA, PIK3R3, CTSK, AKT2, BLNK, TREM2, ACP5, LILRB1, LILRB4, IL1R1, LILRA1, NFATC1, FOSL2, PPP3CA, LILRB2, PIK3R1, RAC1, RELA, MAPK8, IL1B, NFKBIA, CYLD, IFNGR2* |
| KEGG:04210 | Apoptosis | 27 | 0.04485044 | *LMNA, PIK3CA, XIAP, GADD45A, PIK3R3, CTSK, AKT2, HRAS, CTSD, FAS, DIABLO, ITPR1, BBC3, RAF1, CTSL, CTSB, BID, CTSW, CTSS, GZMB, PIK3R1, RELA, TNFSF10, MAPK8, PIDD1, NFKBIA, BCL2L11* |
