# Supplementary material for: Regulation of immune response against third-stage Gnathostoma spinigerum larvae by human genes
Source: Front Immunol. 2023 Aug 3;14:1218965. doi: 10.3389/fimmu.2023.1218965 (PMC10436992; doi:10.3389/fimmu.2023.1218965)
Supplement: Supplementary file 2 [file Table_2.docx]

**Supplementary Table 2**. Reactome analysis in PBMC co-cultured with *G. spinigerum* L3 at day 1 of stimulation.

Eighteen significant pathways related with immune response were significantly expressed at day 1 (*p* value <0.05, FDR <0.05). Comparing among the significant pathways, the immune system pathways (REAC:R-HSA-168256) showed the significant expressed at day 1, with the most member 298 counts

| **Term_ID** | **Term_name** | **Count** | **up-**  **regulate** | **down-regulate** | ***p*-value** |
| --- | --- | --- | --- | --- | --- |
| REAC:R-HSA-73857 | RNA Polymerase II Transcription | - | - | - | - |
| REAC:R-HSA-74160 | Gene expression (Transcription) | 167 | 96 | 71 | 0.0410186 |
| REAC:R-HSA-168256 | Immune System | 298 | 162 | 136 | 1.5863E-08 |
| REAC:R-HSA-212436 | Generic Transcription Pathway | 145 | 83 | 62 | 0.02396573 |
| REAC:R-HSA-380259 | Loss of Nlp from mitotic centrosomes | 13 | 5 | 8 | 0.02731623 |
| REAC:R-HSA-380284 | Loss of proteins required for interphase microtubule organization from the centrosome | 13 | 5 | 8 | 0.02731623 |
| REAC:R-HSA-380320 | Recruitment of NuMA to mitotic centrosomes | 17 | 7 | 10 | 0.04134072 |
| REAC:R-HSA-453274 | Mitotic G2-G2/M phases | 30 | 18 | 12 | 0.04981021 |
| REAC:R-HSA-983169 | Class I MHC mediated antigen processing & presentation | 56 | 29 | 27 | 0.03876962 |
| REAC:R-HSA-913531 | Interferon Signaling | 42 | 30 | 12 | 0.01801564 |
| REAC:R-HSA-1280215 | Cytokine Signaling in Immune system | 120 | 80 | 40 | 0.00062176 |
| REAC:R-HSA-1834949 | Cytosolic sensors of pathogen-associated DNA | 18 | 10 | 8 | 0.04817741 |
| REAC:R-HSA-1852241 | Organelle biogenesis and maintenance | 47 | 19 | 28 | 1.4569E-05 |
| REAC:R-HSA-2565942 | Regulation of PLK1 Activity at G2/M Transition | 15 | 5 | 10 | 0.00323328 |
| REAC:R-HSA-3700989 | Transcriptional Regulation by TP53 | - | - | - | - |
| REAC:R-HSA-5617833 | Cilium Assembly | 32 | 13 | 19 | 0.00011747 |
| REAC:R-HSA-5620912 | Anchoring of the basal body to the plasma membrane | 15 | 5 | 10 | 0.01071944 |
| REAC:R-HSA-6798695 | Neutrophil degranulation | 69 | 28 | 41 | 0.01003457 |
| REAC:R-HSA-6804758 | Regulation of TP53 Activity through Acetylation | 8 | 7 | 1 | 0.00556785 |
| REAC:R-HSA-8854518 | AURKA Activation by TPX2 | 13 | 5 | 8 | 0.02407498 |
