# Supplementary material for: Regulation of immune response against third-stage Gnathostoma spinigerum larvae by human genes
Source: Front Immunol. 2023 Aug 3;14:1218965. doi: 10.3389/fimmu.2023.1218965 (PMC10436992; doi:10.3389/fimmu.2023.1218965)
Supplement: Supplementary file 3 [file Table_3.docx]

**Supplementary Table 3**. Reactome analysis in PBMC co-cultured with *G. spinigerum* L3 at day 3 of culture.

Only 7 pathways were expressed at day 3 (*p* <0.05, FDR <0.05). Comparing among the significant pathways, the immune system pathways (REAC:R-HSA-168256) showed the significant expression at day 3,with the most member 276 counts.

| **Term_ID** | **Term_name** | **Count** | **up-regulate** | **down-regulate** | ***p*-value** |
| --- | --- | --- | --- | --- | --- |
| REAC:R-HSA-73857 | RNA Polymerase II Transcription | 205 | 98 | 107 | 4.70384E-08 |
| REAC:R-HSA-74160 | Gene expression (Transcription) | 232 | 115 | 117 | 3.12274E-10 |
| REAC:R-HSA-168256 | Immune System | 276 | 129 | 147 | 0.002751663 |
| REAC:R-HSA-212436 | Generic Transcription Pathway | 189 | 92 | 97 | 9.93813E-08 |
| REAC:R-HSA-1852241 | Organelle biogenesis and maintenance | 37 | 24 | 13 | 0.034888273 |
| REAC:R-HSA-3700989 | Transcriptional Regulation by TP53 | 63 | 31 | 32 | 0.001625221 |
| REAC:R-HSA-6798695 | Neutrophil degranulation | 81 | 31 | 50 | 0.000197403 |
